# Activity-Based Protein Profiling Reveals Potential Dasatinib Targets in Gastric Cancer

**DOI:** 10.3390/ijms21239276

**Published:** 2020-12-04

**Authors:** Kyoung-Min Choi, Eunji Cho, Geul Bang, Seong-Jae Lee, Boram Kim, Ji-Hee Kim, Seo-Gyu Park, Eun Hee Han, Young-Ho Chung, Jin Young Kim, Eunjung Kim, Jae-Young Kim

**Affiliations:** 1 Graduate School of Analytical Science and Technology (GRAST), Chungnam National University, Daejeon 34134, Korea; kyoungmin11@cnu.ac.kr (K.-M.C.); Ella@genuv.com (E.C.); seongjae.lee10@gmail.com (S.-J.L.); zxcva1750@hanmail.net (B.K.); oliviaj789@naver.com (J.-H.K.); gue95@naver.com (S.-G.P.); chungyh@kbsi.re.kr (Y.-H.C.); 2 Research Center for Bioconvergence Analysis, Korea Basic Science Institute (KBSI), Cheongju 28119, Korea; bangree@kbsi.re.kr (G.B.); heh4285@kbsi.re.kr (E.H.H.); jinyoung@kbsi.re.kr (J.Y.K.); 3 Natural Product Informatics Center, Korea Institute of Science and Technology (KIST), Gangneung 25451, Korea

**Keywords:** dasatinib, gastric cancer, activity-based protein profiling, LC–MS/MS

## Abstract

Dasatinib is a multi-target kinase inhibitor, whose targets include BCR-ABL, SRC family kinases, and various cancer kinases. The elevated SRC activity in gastric cancer (GC) has prompted the need for the therapeutic application of dasatinib in GC. We observed that the efficacy of dasatinib varied with the GC cell lines. The differential effect of dasatinib was not correlated with the basal SRC activity of each cell line. Moreover, the GC cell lines showing the strong antitumor effects of dasatinib were refractory to other SRC inhibitors, i.e., bosutinib and saracatinib, suggesting that unexpected dasatinib’s targets could exist. To profile the targets of dasatinib in GC, we performed activity-based protein profiling (ABPP) via mass spectrometry using a desthiobiotin-ATP probe. We identified 29 and 18 kinases as potential targets in dasatinib-sensitive (SNU-216, MKN-1) and -resistant (SNU-484, SNU-601) cell lines, respectively. The protein–protein interaction mapping of the differential drug targets in dasatinib-sensitive and -resistant GC using the STRING database suggested that dasatinib could target cellular energy homeostasis in the drug-sensitive GC. RNAi screening for identified targets indicated p90RSK could be a novel dasatinib target, which is important for maintaining the viability and motility of GC cells. Further functional validation of dasatinib off-target actions will provide more effective therapeutic options for GC.

## 1. Introduction

Gastric cancer (GC) is the third leading cause of cancer-associated death worldwide, with a particularly high incidence in East Asian countries. The prognosis of patients with metastatic GC is poor [1]. However, the treatment options for non-resectable metastatic GC are currently limited to chemotherapy [2]. Therefore, the development of novel therapeutic strategies for advanced GC is imperative. 

Despite recent advances in targeted cancer therapy [3], only a limited number of GC patients benefit from targeted reagents due to a lack of knowledge regarding the molecular drivers of GC. For example, human epidermal growth factor 2 (HER2) inhibitor, trastuzumab, is the first approved targeted agent for GC patients with HER2 overexpression. However, HER2 is amplified only in 10–25% of GC [4]. Therefore, the identification of new molecular targets for the majority of GC patients is desirable. The new target-based therapy will facilitate the improvement of GC treatment outcomes and increase the survival rate.

SRC is a non-receptor tyrosine kinase whose expression is elevated in multiple cancers. SRC belongs to the family of SRC kinases (SFKs), including nine members (SRC, FYN, LYN, YES, LCK, YRK, BLK, HCK, and FGR). SRC is activated upon stimulation of multiple receptor tyrosine kinases or integrins. The stimulated SRC phosphorylates its downstream signaling pathways implicated in tumorigenesis, including PI3K-AKT, STAT3, RAS-ERK, and FAK. Activation of SRC-mediated signaling contributes to cell proliferation, survival, motility, differentiation, and angiogenesis [5]. Aberrant SRC expression is detected in most GCs and is also associated with cancer progression and poor prognosis [6,7,8,9], providing a clinical rationale for the use of SFK inhibitors as effective targeted therapy for the treatment of GC.

Dasatinib is a potent SFK inhibitor, currently under clinical development for the treatment of multiple solid tumors [10,11]. Given the importance of SFKs in various aspects of tumorigenesis in GC, dasatinib is expected to play a significant role in GC treatment. Previous studies reported that dasatinib has a broad target profile extending beyond the SFK group, indicating that the antitumor efficacy of dasatinib is attributed to its promiscuous nature. Chemical proteomics analyses revealed a broad target spectrum of dasatinib in leukemia [12,13]. Li and colleagues reported that dasatinib binds and targets SFK members as well as a panel of receptor tyrosine kinases (EGFR, ephrin receptors, DDR1) and non-receptor tyrosine kinases (FRK, BRK, ACK) in lung cancer [14]. Recently, target profiling of dasatinib in the context of GC was reported using an immobilized kinase inhibitor coupled with mass spectrometry [15]. However, the mechanism of action of dasatinib in GC has yet to be elucidated.

In this study, we investigated the mechanisms of action of dasatinib in GC harnessing mass spectrometry-based activity-based protein profiling (ABPP). We first investigated various phenotypic effects of dasatinib in multiple gastric cancer cells and compared the effects of treatment with other SFK inhibitors. The results raised the possibility of potential off-target actions of dasatinib. Our ABPP study revealed differential target profiles of dasatinib between dasatinib-sensitive and -resistant GC cells. The protein–protein interaction mapping of differential dasatinib target profiles revealed that dasatinib could target cellular energy homeostasis in sensitive GC cells. Furthermore, RNAi screening for dasatinib targets identified p90RSK as a novel dasatinib target in GC. Pharmacological inhibition of p90RSK reduced GC cell viability and migration, suggesting the antitumor effect of dasatinib could be attributed to its inhibitory effect on p90RSK.

## 2. Results and Discussion

### 2.1. Phenotypic Effects of Dasatinib in GC Cells

**Figure 1:**
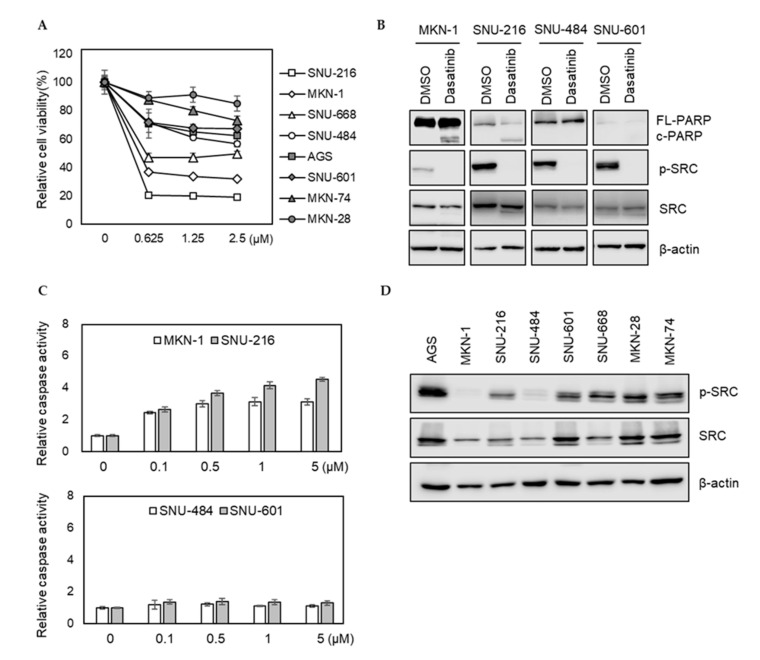
Phenotypic effects of dasatinib in gastric cancer (GC) cell lines. (**A**) Relative cell viability after dasatinib treatment in GC cells. Cells were treated with different doses of dasatinib (0 to 2.5 μM) for 72 h, and the cell viability was assessed by MTT assay. Error bars represent standard deviation from representative triplicate experiments from at least three experiments that showed similar results. (**B**) Cells were treated with 500 nM of dasatinib for 48 h, and PARP cleavage and SRC levels were analyzed by Western blot. FL-PARP: full length PARP; c-PARP: cleaved PARP; p-SRC: phospho-SRC (Y416). (**C**) Cells were treated with different doses of dasatinib (0 to 5 μM) for 24 h, then caspase activity was analyzed using a Caspase Glo 3/7 assay kit. Relative caspase activities are shown. Error bars represent standard deviation from representative triplicate experiments from at least three experiments that showed similar results. (**D**) Basal phospho-SRC (Y416) and total SRC levels in gastric cancer cell lines were determined by Western blot.

We investigated how eight different GC cell lines (AGS, MKN-1, MKN-28, MKN-74, SNU-216, SNU-484, SNU-601, and SNU-668) responded to dasatinib. Dasatinib markedly reduced the viabilities of MKN-1 and SNU-216 cells from the lowest test dose (0.625 μM). However, it showed a modest effect on other GC cells (Figure 1A). To further characterize the effect of dasatinib in GC, we investigated whether dasatinib induced apoptosis in dasatinib-sensitive cell lines. Consistent with the cell viability results, dasatinib induced PARP cleavage and caspase activation in dasatinib-sensitive MKN-1 and SNU-216 cells; however, it failed to induce apoptosis in SNU-484 and SNU-601 cells in which dasatinib showed marginal effects on cell viability (Figure 1B,C). To investigate whether this heterogeneous response to dasatinib is related to basal SRC activity, we examined SRC tyrosine phosphorylation (Y416) in GC cells. The basal SRC phosphorylation at tyrosine 416 residue in dasatinib-sensitive MKN-1 was barely detectable. The SRC phosphorylation of SNU-216, another dasatinib-sensitive cell line, was comparable to other GC lines, where dasatinib showed modest effects (Figure 1D). These results suggest that dasatinib sensitivity is not associated with basal SRC activity, which raises the possibility that dasatinib exerted an antitumor effect by inhibiting other targets in GC cells.

### 2.2. Differential Antitumor Effect of Dasatinib Compared with Other SFK Inhibitors, Saracatinib and Bosutinib, in GC Cells

**Figure 2:**
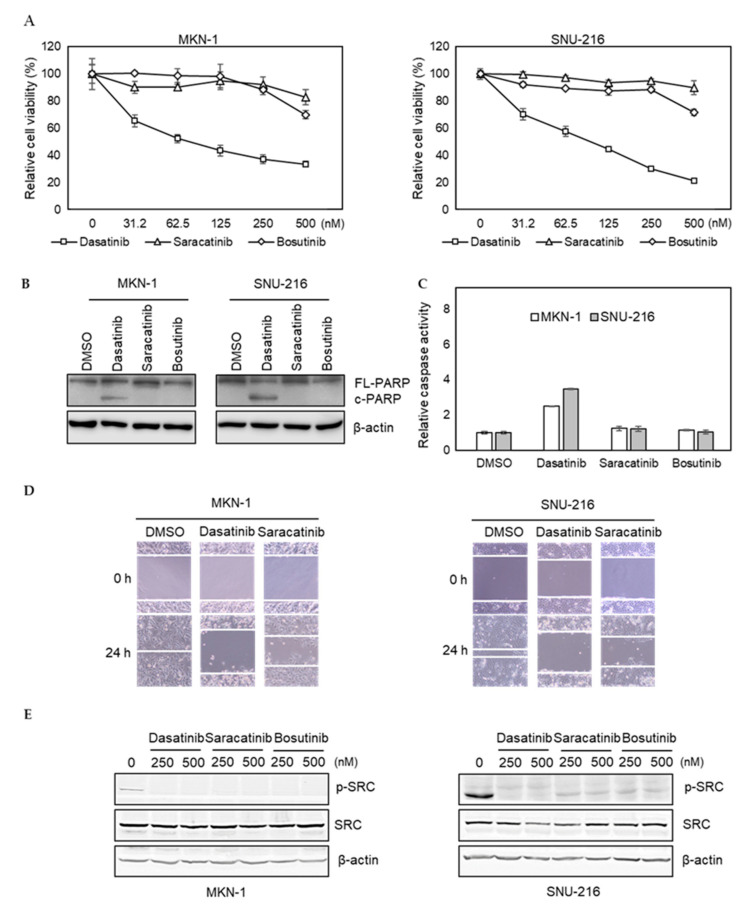
Comparison of dasatinib effect with other SRC inhibitors in GC cell lines. (**A**) Relative viabilities of GC cells in response to dasatinib, saracatinib, and bosutinib. Cells were treated with different doses of drugs (0 to 2.5 μM) for 72 h, and the cell viability was assessed by MTT assay. Error bars represent standard deviation from representative triplicate experiments from at least three experiments that showed similar results. (**B**) Cells were treated with 500 nM of drugs for 48 h, and PARP cleavage and SRC levels were analyzed by Western blotting. FL-PARP: full length PARP; c-PARP: cleaved PARP; p-SRC: phospho-SRC (Y416). (**C**) Cells were treated with 500 nM of drugs for 24 h, then caspase activity was analyzed using a Caspase Glo 3/7 assay kit. Relative caspase activities are shown. Error bars represent standard deviation from representative triplicate experiments from at least three experiments that showed similar results. (**D**) Cells were treated with vehicle control (DMSO) or 250 nM of drugs for 24 h, then cell migration was analyzed by wound healing assay. (**E**) Cells were treated with 250 nM or 500 nM of drugs for 1 h, and phospho- (Y416) or total SRC levels were analyzed by Western blotting.

To further investigate potential off-target effects of dasatinib in GC, we tested whether saracatinib and bosutinib, other SRC inhibitors, showed similar efficacy. We found that the efficacy of saracatinib and bosutinib was not as pronounced as dasatinib in dasatinib-sensitive MKN-1 and SNU-216 cells (Figure 2A). Likewise, these two other SRC inhibitors failed to induce PARP cleavage and caspase activation in dasatinib-sensitive GC cells (Figure 2B,C). Furthermore, in contrast to saracatinib, dasatinib significantly reduced the migration of dasatinib-sensitive GC cells (Figure 2D). We confirmed that the ability of these three inhibitors to inhibit the activity of SRC was similar, indicating that the inability of saracatinib and bosutinib to inhibit the proliferation and migration of GC cells was not due to incomplete inhibition of SRC (Figure 2E). Taken together, these results suggest that dasatinib inhibits additional unknown targets, which are important for the viability and migration of GC cells.

### 2.3. Activity-Based Protein Profiling of Dasatinib Targets in GC Cells

**Figure 3:**
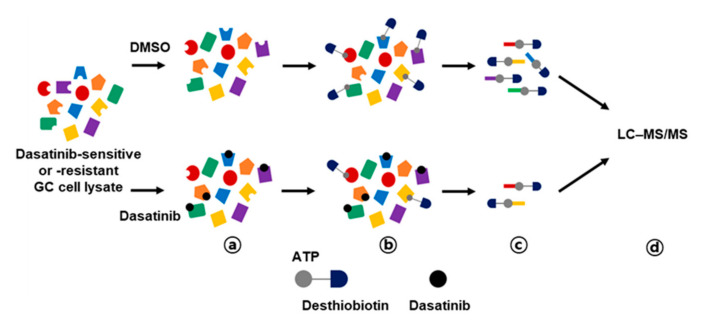
Workflow for dasatinib target profiling via ATP probe-based activity-based protein profiling (ABPP) in GC. Cell lysates from dasatinib-sensitive (MKN-1 and SNU-216) and -resistant (SNU-484 and SNU-601) GC cell lines were preincubated with vehicle control (DMSO) or 10 μM of dasatinib (a), then reacted with desthiobiotin-ATP probe (b). Subsequently, cell lysates were trypsin digested and labeled peptides were purified by streptavidin agarose beads (c). Probe-labeled peptides were analyzed by LC–MS/MS (d).

Activity-based protein profiling (ABPP) employs chemical probes that bind to active sites of enzymes, enabling quantification of individual enzyme activity [16]. In this study, we performed ABPP using desthiobiotin-ATP probes in conjunction with LC–MS/MS to enrich dasatinib kinase targets that could be important for the survival of GC cells. This approach has been used for target profiling of kinase inhibitor drugs to identify actionable drug targets in small cell lung cancer [17]. We hypothesized that differential target profiling of dasatinib-sensitive and -resistant GC cells could provide novel insights into survival kinases in GC cells. Thus, we selected two dasatinib-sensitive GC cells (MKN-1 and SNU-216) and two dasatinib-resistant GC cells (SNU-484 and SNU-601). The ABPP approach is outlined in Figure 3. Briefly, the desthiobiotin-ATP probe competition reaction was performed after preincubation of cell lysate with dasatinib or vehicle control (DMSO), then the probe-labeled lysates were digested and enriched with streptavidin beads. The enriched peptides were analyzed in LC–MS/MS and quantitated using MaxQuant software. The desthiobiotin labeling and dasatinib competition were validated by Western blotting for SRC, which is the primary target of dasatinib (Supplementary Figure S1).

**Figure 4:**
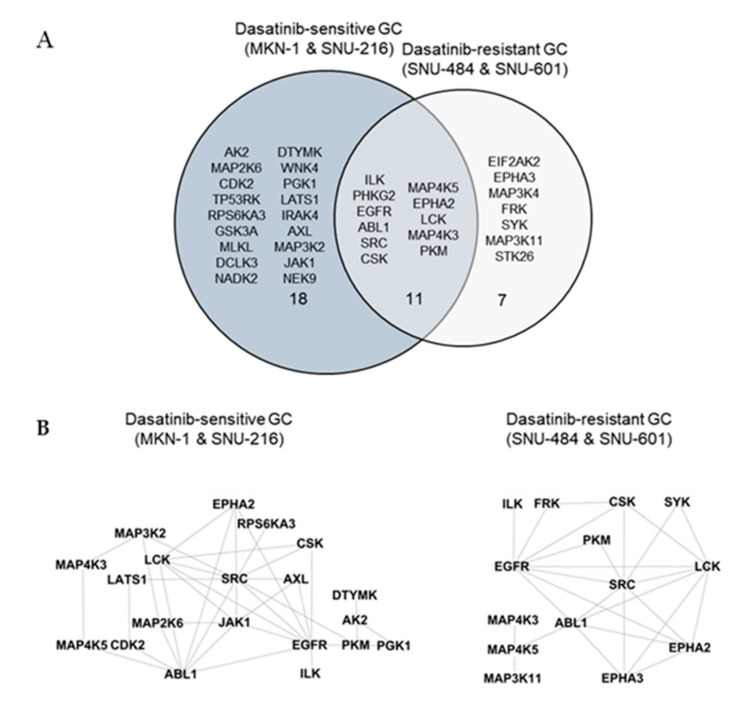

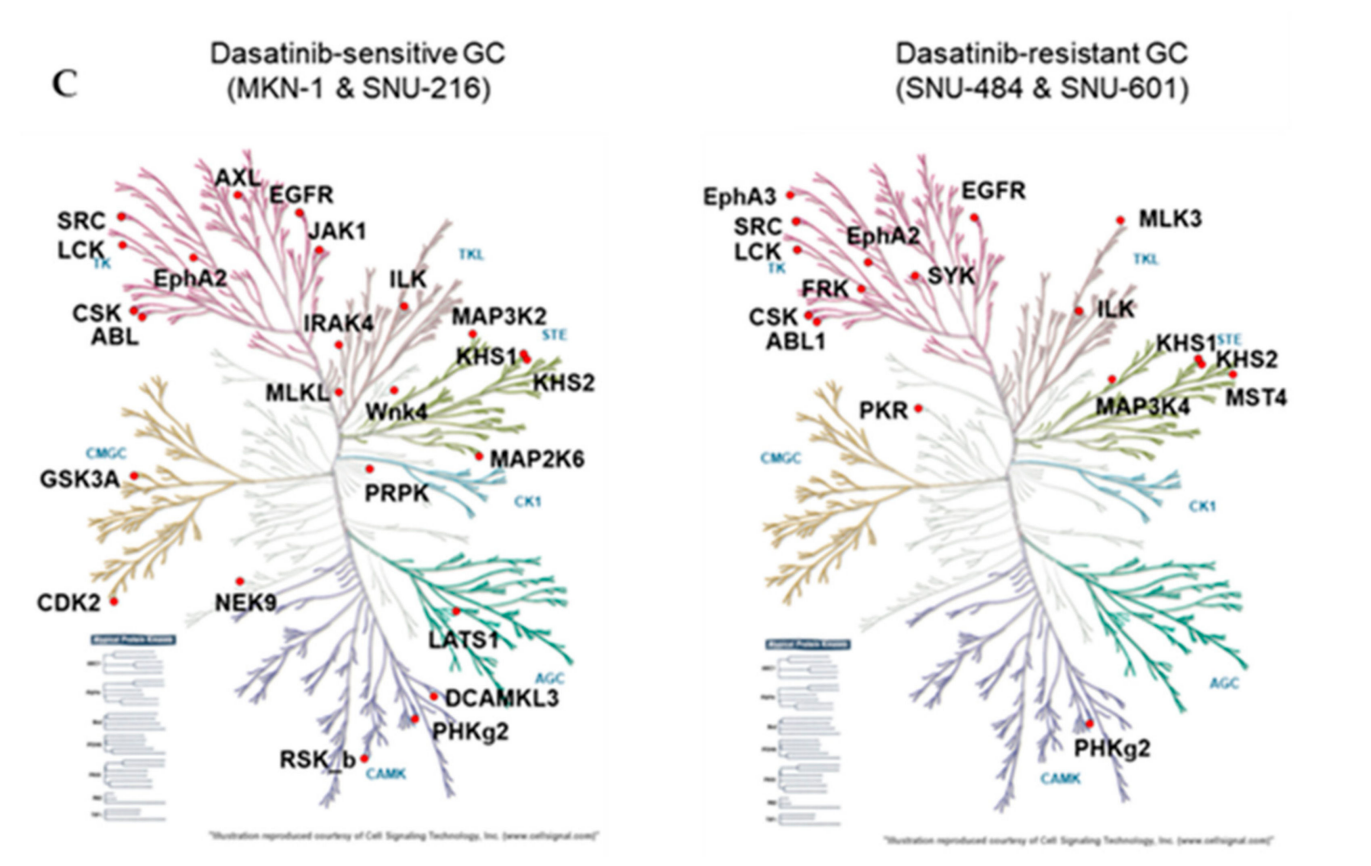
Differential dasatinib target profiling between dasatinib-sensitive and -resistant GC. (**A**) Venn diagram showing dasatinib target kinases in dasatinib-sensitive and -resistant GC. (**B**) Protein–protein interaction networks for dasatinib target kinases identified from dasatinib-sensitive and -resistant GC. The STRING database was used to map protein–protein interactions and Cytoscape software was used to illustrate the resulting network. (**C**) Dasatinib target kinases in sensitive and resistant GC cells illustrated in the kinome tree.

We identified a total of 246 peptides that corresponded to 118 kinases. Among them, the ATP labeling of 44 peptides corresponding to 36 kinases was reduced (competed) >50% by dasatinib. We identified 29 protein kinases targeted by dasatinib in dasatinib-sensitive GC cells (MKN-1 and SNU-216) and 18 protein kinases in dasatinib-resistant GC cells (Supplementary Table S1 and Figure 4A). The majority of dasatinib target kinases identified in both dasatinib-sensitive and -resistant GC cells were reportedly dasatinib targets, which include ABL, SRC family kinases (SRC, LCK), and receptor tyrosine kinases (EGFR, EPHA2) [18,19]. Next, we constructed a network view based on protein–protein interactions between dasatinib target kinases using the STRING database in dasatinib-sensitive and -resistant GC, respectively (Figure 4B). The network view showed that SRC, the primary target of dasatinib, is located in the center node linked to receptor tyrosine kinases (EGFR, ephrin receptors) and another SRC family kinase (LCK) in both dasatinib-sensitive and -resistant GC cells. Notably, we observed a cluster of glycolytic kinases (phosphoglycerate kinase 1 (PGK1) and pyruvate kinase M (PKM)), and kinases involved in nucleotide metabolism (adenylate kinase (AK2), thymidylate kinase (DTYMK)) in dasatinib-sensitive cells. This result suggests that dasatinib could target cellular energy homeostasis in sensitive GC cells. Furthermore, we marked dasatinib target kinases in the kinome tree in order to acquire broader insights into the mechanism of action of dasatinib in GC cells (Figure 4C). Kinases, which belong to groups of tyrosine kinase (TK), tyrosine kinase-like (TKL), and homologs of yeast sterile 7, 11, 20 kinases (STE), were covered in both dasatinib-sensitive and -resistant GC cells. Notably, kinases that belong to CDK, MAPK, GSK3, CLK families (CMGC), casein kinase 1 (CK1), and PKA, PKG, PKC (AGC) subfamilies were explicitly enriched in dasatinib-sensitive GC cells. Among these kinases, the cyclin-dependent kinase 2 (CDK2) was worth investigating further. CDK2 is a serine/threonine-protein kinase that regulates cell division, including DNA synthesis, G1-S, and G2 progression [20]. Previous studies reported that CDK2 inhibition reduced cancer proliferation and cell cycle arrest in the breast, colon cancer, and cholangiocarcinoma [21,22,23,24]. Interestingly, dasatinib induced cell cycle arrest (G1 arrest) in non-small cell lung cancer (NSCLC), head and neck squamous cell carcinoma (HNSCC), and leukemia cell lines [25,26], suggesting that dasatinib negatively regulated the cell cycle machinery. Notably, Shi and colleagues reported CDK2 as a potential dasatinib target in leukemia [27]. Thus, it is possible that CDK2 is one of dasatinib target kinases that is important in maintaining GC cell viability.

Notably, IL-1 receptor-associated kinase 4 (IRAK4) was identified as a dasatinib target in dasatinib-sensitive GC cells. IRAK4, a serine/threonine kinase involved in the signaling cascade mediated by toll-like receptor (TLR) and interleukin-1(IL-1) receptor, plays a key role in NF-kB activation in innate immune response [28]. Recently, the importance of IRAK4 as a cancer therapeutic target has been recognized. IRAK4-mediated NF-κB is implicated in tumorigenesis and chemoresistance of pancreatic and colorectal cancers [29,30]. Few studies have reported the importance of IRAK4 in GC. However, NF-kB induced by chronic *Helicobacter pylori* infection contributes to GC tumorigenesis via the production of proinflammatory cytokines/chemokines, growth factors, angiogenesis regulators, and metalloproteinases [31]. Given the importance of IRAK4 in TLR-mediated innate immune signaling, targeting IRAK4-mediated NF-kB signaling could be a promising therapeutic option in GC. Our results suggest that dasatinib not only inhibits oncogene-driven tyrosine kinase signaling but may also impair tumor-promoting innate immune signaling by targeting IRAK4 in GC. Although the primary dasatinib targets are tyrosine kinases, Shi and colleagues reported that several serine/threonine kinases were targeted by dasatinib [27]. Thus, it is possible that IRAK4 is another serine/threonine kinase target of dasatinib.

### 2.4. Dasatinib Inhibits p90RSK Activity and Pharmacological Inhibition of p90RSK Impairs GC Cell Viability and Migration

**Figure 5:**
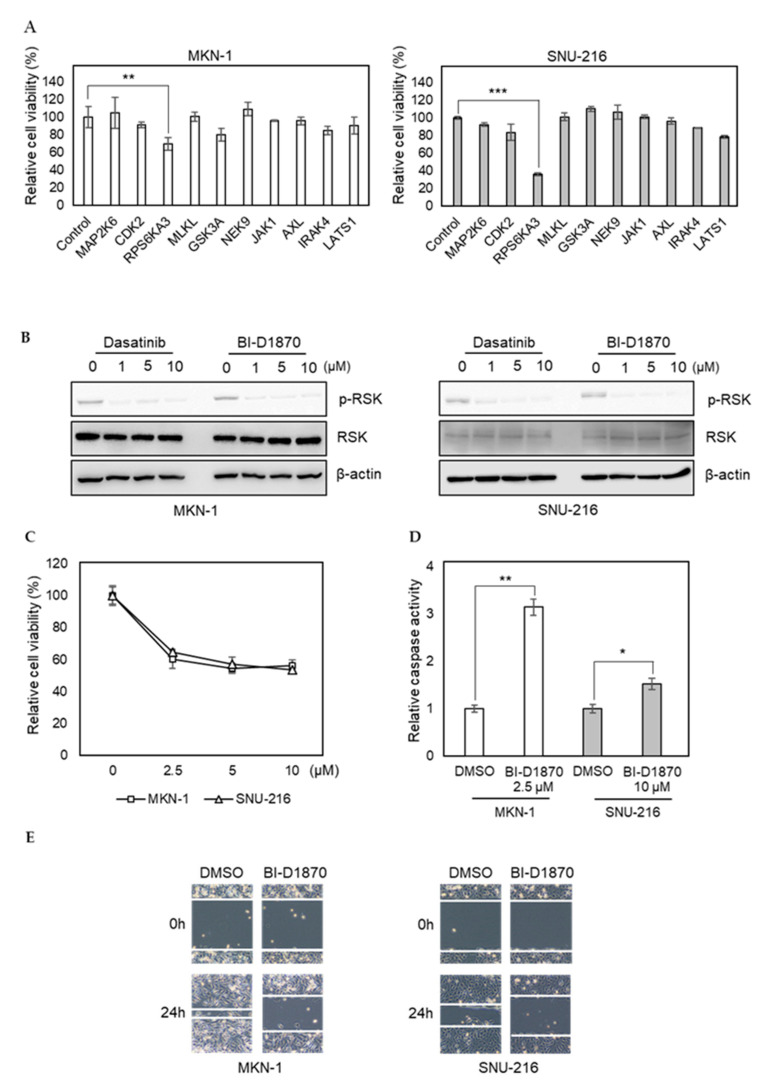
RNAi screen identifies p90RSK as a novel dasatinib target and it is important for GC cell viability and motility. (**A**) Relative viabilities of GC cells in response to siRNA transfection. The cell viability was assessed by MTT assay. Error bars represent standard deviation from representative triplicate experiments from two experiments that showed similar results. ** *p* < 0.01, *** *p* < 0.001. (**B**) Cells were treated with different doses of drugs (0 to 10 µM) for 1 h, and phospho-RSK (T359) or total RSK levels were analyzed by Western blotting. (**C**) Relative viabilities of GC cells in response to BI-D1870 exposure. Cells were treated with different doses of drugs (0 to 10 µM) for 72 h, and the cell viability was assessed by MTT assay. Error bars represent standard deviation from representative triplicate experiments from at least three experiments that showed similar results. (**D**) Cells were treated with 10 µM of BI-D1870 for 24 h, then caspase activity was analyzed using a Caspase Glo 3/7 assay kit. Relative caspase activities are shown. Error bars represent standard deviation from representative triplicate experiments from two experiments that showed similar results. * *p* < 0.05, ** *p* < 0.01. (**E**) Cells were treated with vehicle control (DMSO) or 5 µM of BI-D1870 for 24 h, then cell migration was analyzed by wound healing assay.

To investigate the importance of newly identified dasatinib targets in GC, we transfected MKN-1 and SNU-216 cells with siRNAs corresponding to selected kinases identified in dasatinib-sensitive cells only (MAP2K6, CDK2, RPS6KA3, MLKL, GSK3A, NEK9, JAK1, AXL, IRAK4, and LATS1). Our selection was based on the functional relevance of targets in cancer phenotypes, such as proliferation and metastasis. We found knocking down RPS6KA3 significantly reduced cell viability, whereas the effects of other siRNAs were marginal (Figure 5A). RPS6KA3 belongs to the p90 ribosomal S6 kinase (p90RSK) family, which acts downstream of ERK signaling and mediates mitogen-induced cellular proliferation, survival, and differentiation [32,33,34]. We found that dasatinib could inhibit the activating phosphorylation of p90RSK in GC cells (Figure 5B). To further investigate the role of p90RSK as a novel dasatinib target in GC, we tested whether pharmacological inhibition of p90RSK could recapitulate dasatinib phenotypes. BI-D1870, a p90RSK inhibitor, could reduce cell viability of MKN-1 and SNU-216 as well as induce caspase activation (Figure 5C,D). Moreover, BI-D1870 could reduce the migration of these GC cells as well (Figure 5E). These results suggest that antitumor effect of dasatinib could be attributed to its inhibitory effect on p90RSK.

In this study, we report the heterogeneous response to dasatinib in a panel of GC cell lines. We found that SRC was not the primary target contributing to dasatinib sensitivity in GC. The mass spectrometry-based approach revealed differential dasatinib targets in dasatinib-sensitive and -resistant GC cells. Protein–protein interaction mapping of the differential dasatinib targets revealed that dasatinib could modulate cellular energy homeostasis in sensitive GC cells. RNAi screening for newly identified dasatinib targets identified p90RSK as a novel dasatinib target, which is important for GC cell viability and migration. Further functional validation of newly identified dasatinib targets will provide novel insights into targeted therapies for GC.

## 3. Materials and Methods

### 3.1. Cell Culture

All GC cell lines were obtained from the Korean Cell Line Bank. Cells were cultured in RPMI1640 medium containing 10% FBS and 1% antibiotic-antimycotic (HyClone, Logan, UT, USA), and maintained at 37 °C in an atmosphere with 5% CO_2_.

### 3.2. Chemicals and Antibodies

The SRC inhibitors (dasatinib, bosutinib, and saracatinib) were purchased from Selleckchem (Houston, TX, USA). All chemicals were dissolved in dimethyl sulfoxide (DMSO) and aliquots were stored at −80 °C. Primary antibodies were purchased from Cell Signaling Technology (Danvers, MA, USA) and horseradish peroxidase (HRP)-conjugated secondary antibodies were purchased from Thermo Fisher Scientific (Waltham, MA, USA). 

### 3.3. Cell Viability Assay

Cells were seeded in 96-well plates at 20–30% density, followed by drug treatment the next day. After 3 days of incubation, 10 µL of 5 mg/mL MTT solution were added to each well (Sigma-Aldrich, St. Louis, MO, USA) and cells were incubated for 2 h. The cell culture medium was removed and formazan was dissolved by the addition of 60 µL of DMSO. Cell viability was quantified by microplate reader (VersaMax, Molecular Devices, San Jose, CA, USA) at 570 nm.

### 3.4. Western Blot

Cells were lysed in NETN buffer (100 mM NaCl, 20 mM Tris pH 8.0, 0.5 mM EDTA, 0.5% NP-40) supplemented with protease and phosphatase inhibitor cocktail (GenDepot, Baker, TX, USA). Approximately 50 μg of protein were resolved via 8% SDS-PAGE gel and transferred to a nitrocellulose membrane. The membrane was blocked with 5% skim milk in TBST and incubated with primary antibodies at 4 °C overnight. After washing with PBST three times, the membrane was incubated with HRP-conjugated secondary antibodies at room temperature for 1 h. The Fusion Solo Chemidoc system (Vilber, Marne-La-Vallée, Collégien, France) was used to detect chemiluminescence.

### 3.5. Caspase 3/7 Assay

Cells were seeded onto a 96-well plate at 7 × 10^3^ cells per well. Cells were treated with drugs the following day for 24 h, followed by the addition of Caspase-Glo^®^ 3/7 Reagent (Promega, Madison, WI, USA) and analysis of caspase activity according to the manufacturer’s protocol.

### 3.6. Wound Healing Assay

Wounds were created using silicone inserts (Ibidi, Gräfelfing, BY, Germany) according to the manufacturer’s protocol. Briefly, inserts were placed on a 6-well plate and cells were seeded to generate a confluent monolayer. On the following day, these inserts were removed and the cells were washed to remove floating cells. Images were taken immediately after insert removal (0 h) and 24 h later with a phase-contrast microscope (Olympus, Shinjuku, Tokyo, Japan).

### 3.7. siRNA Screening

The reverse transfection was performed using Lipofectamine RNAiMAX according to the manufacturer’s protocol (Thermo Fisher Scientific, Waltham, MA, USA). Briefly, 4 pmol of siRNA were mixed with 0.4 µL of RNAiMAX transfection reagent in 20 µL of Opti-MEM medium (Thermo Fisher Scientific, Waltham, MA, USA) in a 96-well plate. After 30 min incubation, 3000 cells resuspended in 80 µL of culture media were added into each well (40 nM final siRNA concentration). After 4 days of incubation, cell viability was analyzed by MTT assay. The siRNA sequences used for this study can be found in Supplementary Table S2.

### 3.8. Activity-Based Protein Profiling

Cell lysate preparation as well as labeling and enrichment of ATP-binding proteome were conducted according to the protocols described in the Pierce Kinase Enrichment kit and ActivX probes (Thermo Fisher Scientific, Waltham, MA, USA). Briefly, cell pellets were harvested and lysed with IP lysis buffer supplemented with protease and phosphatase inhibitors. The cell lysate was desalted using Zeba Spin Desalting Columns (Thermo Fisher Scientific, Waltham, MA, USA). For drug profiling, 10 μL of 1M MgCl_2_ were added to 1 mg/500 µL of desalted lysate and incubated for 10 min. The lysates were preincubated with dasatinib or DMSO for 15 min at 10 µM concentration. Subsequently, a desthiobiotin-ATP probe competition reaction was performed for 15 min at 10 μM concentration. The probe-labeled lysates were denatured with 5 M urea, and reduced and alkylated via treatment with 5 mM DTT at 65 °C for 30 min and 40 mM iodoacetamide at room temperature for 30 min, respectively. The samples were again desalted using Zeba Spin Desalting Columns and digested with 20 μg of MS-grade trypsin. The labeled tryptic peptides were enriched with a 50 μL slurry of high capacity streptavidin agarose resin for 1 h at room temperature. Beads were washed three times each with 500 µL of IP lysis buffer, PBS, and LC-grade water. The labeled peptides were eluted with elution buffer (50% ACN, 0.1% TFA), and lyophilized. The lyophilized sample was resuspended in an injection buffer (50% ACN, 0.1% TFA) for LC–MS/MS analysis. The analysis was performed in triplicate with each cell line (3 DMSO vs. 3 dasatinib).

### 3.9. D-LC–MS/MS

Peptides were analyzed using an LC–MS/MS system consisting of an Easy nLC 1000 (Thermo Fisher Scientific) and an Orbitrap Elite mass spectrometer (Thermo Fisher Scientific) equipped with a nano-electrospray source (EASY-Spray Sources, Thermo Fisher Scientific). Peptides were trapped in a 75 μm × 2 cm C18 precolumn (nanoViper, Acclaim PepMap100, Thermo Fisher Scientific, Waltham, MA, USA) before being separated in an analytical C18 column (75 μm × 50 cm PepMap RSLC, Thermo Fisher Scientific, Waltham, MA, USA) at a flow rate of 250 nL/min. The mobile phases A and B were composed of 0% and 100% acetonitrile (Honeywell Burdick & Jackson, Muskegon, MI, USA) containing 0.1% formic acid (Sigma-Aldrich, St. Louis, MO, USA), respectively. The LC gradient began with 5% B and 5% B for 3 min, 25% B for 73 min, to 40% B for 20 min, to 95% B for 1 min, and remained at 95% B over 8 min. Finally, it was ramped to 5% B for another 5 min. The column was reequilibrated with 5% B for 10 min before the next run. The voltage applied to produce an electrospray was 2000 V. During the chromatographic separation, the Orbitrap Elite was operated in data-dependent mode, automatically switching between MS1 and MS2. The MS data were acquired using the following parameters: full-scan MS1 spectra (400–2000 *m/z*) were acquired in the Orbitrap for a maximum ion injection time of 200 ms at a resolution of 120,000 and automatic gain control (AGC) target value of 4 × 10^5^. MS2 spectra were acquired in the Orbitrap mass analyzer at a resolution of 30,000 with high energy collision dissociation (HCD) of 27% normalized collision energy and AGC target value of 5 × 10^4^ with maximum ion injection time of 20 ms. Previously fragmented ions were excluded for 60 s.

### 3.10. Data Analysis

Mass spectrometry data were analyzed using MaxQuant software (v1.5.3.8, https://www.maxquant.org). Desthiobiotin was selected as the search parameter and default parameters were applied for other settings. MS/MS data were searched against the SwissProt human database. The Normalizer tool (http://quantitativeproteomics.org/normalyzer; [35]) was used to normalize the peptide intensity of each sample. Kinases were selected under stringent quality control. As a part of the quality control mechanism, the labeled peptides measuring less than the 3 samples (out of the total 6 samples per cell line) were removed for further analysis. Dasatinib target kinases were defined when dasatinib inhibited the labeled peptide intensity by more than 50%. Protein–protein interaction networks for dasatinib target kinases were analyzed using the STRING database (https://string-db.org), and the resulting networks were visualized using the Cytoscape program (http://cytoscape.org). The KinHub tool (http://kinhub.org) was used to map dasatinib targets on the kinome tree.

## Supplementary Materials

Supplementary materials can be found at https://www.mdpi.com/1422-0067/21/23/9276/s1.

## Abbreviations

ABPP	activity-based protein profiling
AK2	adenylate kinase
AKT	protein kinase b
ATP	adenosine triphosphate
BCR	breakpoint cluster region protein
CDK	cyclin-dependent kinase
CK1	casein kinase 1
CMGC	clk families
DMSO	dimethyl sulfoxide
DTYMK	thymidylate kinase
ERK	extracellular-signal-regulated kinase
FAK	focal adhesion kinase
GC	gastric cancer
GSK3	glycogen synthase kinase 3
HER2	human epidermal growth factor 2
HNSCC	head and neck squamous cell carcinoma
IL-1	interleukin-1
IRAK4	il-1 receptor-associated kinase 4
LC-1 rec	liquid chromatography with tandem mass spectrometry
MAPK	mitogen-activated protein kinase
MTT	(3-(4,5-dimethylthiazol-2-yl)-2,5-diphenyltetrazolium bromide)
NF-κB	nuclear factor kappa-light-chain-enhancer of activated b cells
NSCLC	non-small cell lung cancer
p90RSK	90 kda ribosomal s6 kinases
PARP	poly(adp-ribose) polymerase
PGK1	phosphoglycerate kinase 1
PI3K	phosphoinositide 3-kinases
PKA	protein kinase a
PKC	protein kinase c
PKG	cgmp-dependent protein kinase
PKM	pyruvate kinase m
RPS6KA3	ribosomal protein s6 kinase a3
SFKs	src-family tyrosine kinases
STAT3	signal transducer and activator of transcription 3
STE	homologs of yeast sterile 7, 11, 20 kinases
STRING	search tool for the retrieval of interacting genes/proteins
TK	tyrosine kinase
TKL	tyrosine kinase-like
TLR	toll-like receptor
